# Efficacy of a mineralized collagen bone-grafting material for peri-implant bone defect reconstruction in mini pigs

**DOI:** 10.1093/rb/rby029

**Published:** 2019-03-21

**Authors:** Enbo Wang, Jianmin Han, Xuehui Zhang, Yuhan Wu, Xu-Liang Deng

**Affiliations:** 1 Department of Oral and Maxillofacial Surgery, Peking University School and Hospital of Stomatology, 22 Zhongguancun South Street, Haidian District, Beijing, China; 2 Department of Dental Materials, Peking University School and Hospital of Stomatology, 22 Zhongguancun South Street, Haidian District, Beijing, China; 3 Department of Geriatric Dentistry, Peking University School and Hospital of Stomatology, 22 Zhongguancun South Street, Haidian District, Beijing, China

**Keywords:** dental implant, bone-grafting material, mineralized collagen

## Abstract

The mechanism of the mineralization process induced by natural mineralized collagen (MC) has been investigated for decades. The purpose of this study was to investigate the efficacy of self-assembled MC for peri-implant bone defect reconstruction in a mini pig. A standardized peri-implant bone defect model was created using 14 mini pig mandibles. Two materials were evaluated, i.e. a mixture of hydroxyapatite and collagen (Type A, TA), and self-assembled MC (Type B, TB). Bio-Oss (BO) and untreated (blank control, BC) groups were used as controls. After 3- and 6-month healing periods, the mini pigs were sacrificed for histomorphometric and microcomputed tomography analysis. After 3 months of healing, the average alveolar ridge height was 3.27 ± 1.57 mm for group TA, 3.28 ± 2.02 mm for group TB and 3.37 ± 1.09 mm for group BO, while group BC showed the lowest height of 2.68 ± 0.47 mm. After 6 months of healing, the average alveolar ridge height was 2.64 ± 1.13 mm for group TA, 4.31 ± 1.80 mm for group TB and 3.87 ± 1.38 mm for group BO, while group BC showed the lowest height of 2.48 ± 1.80 mm. The experimental groups and control group showed similar bone volume density, bone complexity and histological reaction. The self-assembled MC (Type B) stimulated new bone formation in the reconstruction of deficient alveolar ridges around the dental implant; it also displayed excellent clinical operability compared with bone grafts without collagen.

## Introduction

Dental implant restoration is becoming increasingly popular for tooth-loss patients. Sufficient bone volume is needed to guarantee the long-term success of dental implant placement [1, 2]. However, the outcomes of localized and generalized alveolar bone defects after tooth loss can vary according to dental infections, alveolar trauma, extractions and periodontal disease [3–5]. Reconstruction of resorbed alveolar ridges has been a goal and challenge for clinicians to optimize the outcomes of oral implant placements. Various surgical approaches have been used to enhance the alveolar bone volume, including but not limited to ridge splitting, distraction osteogenesis, onlay and particulate bone-grafting. Combining bone graft materials with carrier membranes is often used to obtain adequate bone height, bone width, and ridge contour for small or peri-implant defects.

There are four major types of graft materials commonly used for alveolar regeneration application, i.e. autografts, allografts, xenografts and alloplasts. Although an autogenous bone graft obtained from the same individual for ridge repair is considered the gold standard [6–8], the sources are limited. Allogenic bone graft substitutes are generally sourced from cadavers, and are often available in a demineralized, freeze-dried form. Although allogenic materials yield good clinical results, there is a risk of disease transmission, and there is also a possibility of rejection reactions. Xenogenic bone is derived from nonhuman sources (e.g. bovine, porcine and equine bone,). However, xenogenic bone resorbs slowly and also carries a risk of disease transmission and potential immunological reactions. Alloplastic bone graft substitutes manufactured from mineral raw materials are of increasing interest because their compositions can be precisely adjusted, their resource is without limited, there is no risk of disease pathogen transmission and there are no ethical concerns [9]. The most commonly used alloplastic materials are hydroxyapatite (HA), tricalcium phosphate (TCP), various biphasic combinations of TCP and HA, and bioactive glasses [10]. However, alloplasts have no osteoinductive or osteogenic potential, some studies show slow bone formation *in vivo* and the ostointegration is not always ideal [11].

Cui *et al.* [12] designed and prepared biomimetic mineralized collagen (MC) nanofibrils, which resembled natural bone in terms of both composition and nanostructure. Previous research demonstrated that MC can promote cell proliferation and osteogenic differentiation of human mesenchymal stem cells. Microarray analysis showed that MC was conducive to the expression of osteogenesis-related genes, such as BMP-2, COL1A1 and CTSK, and stimulated osteogenic differentiation, such as by osteoblast differentiation and skeletal system development pathways [13]. Additionally, MC has been used to repair critical-sized defects in the long bones [14]. However, the efficiency of self-assembled MC for peri-implant bone defect reconstruction remains unknown. Therefore, the aim of the present study was to evaluate the clinical efficacy of self-assembled MC grafting materials in the alveolar ridge of mini pig. We hypothesis that there is no significant difference for the tested materials.

## Experimental

### Materials

The Type A (TA) and Type B (TB) materials were prepared and provided by the School of Materials Science and Engineering of Tsinghua University and Beijing Allgens Medical Technology Co., Ltd., respectively. The TA material was a 7/3 mixture of HA and collagen, with a partially MC component. The TB material also had a HA/collagen ratio of 7/3 with MC. The nanosized HA was periodically and orderly arranged between collagen molecules, which self-assembled to form the basic structural units of the MC. Bio-Oss (BO), which is bovine bone calcined at high temperature, was purchased from Geistlich Pharma AG (Switzerland). It was free of all organic components and was used in the control group. The blank control (BC) group contained only bone defects with a collagen membrane (Bio-Gide; Geistlich), and did not contain any graft-filling material.

### Surgical procedure

#### Tooth extraction

Fourteen female mini pigs, aged 12 months and with an average body weight of 35 kg, were used in the present study. The study protocol was approved by the Animal Care and Use Committee of Peking University. The animals were anesthetized by injection of pentobarbital sodium (30 mg/kg body weight). After disinfection of the surgical site with chlorohexidine solution (0.2%), local infiltration anesthesia was given. The continuous incision in the gingival crevicular was conducted, the full thickness mucoperiosteal flap was carefully elevated, both side premolars and first molar of lower mandible were sectioned and separated using a high-speed-turbine tooth drill, and carefully extracted from both hemimandibles. The teeth extraction socket was carefully examined to ensure there is no root remnants were left. The mucoperiosteal flap was closely sutured with absorbable suture line.

#### Defect creation, implant insertion and ridge augmentation

**Figure 1:**
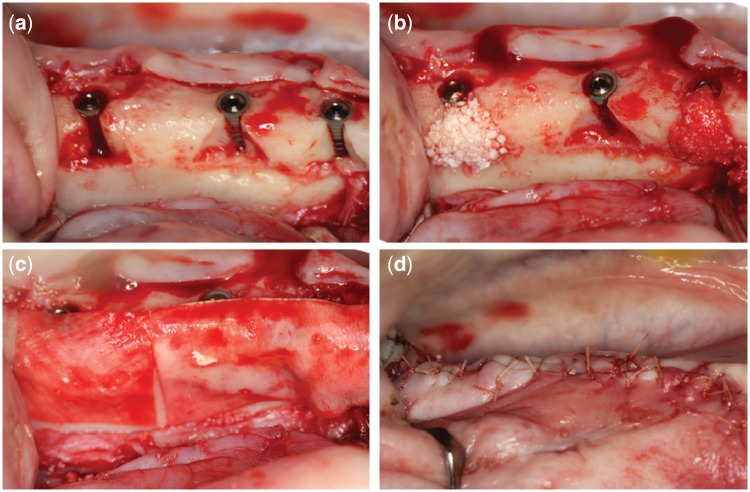
The process of bone defect creation, implant insertion and ridge augmentation. (a) Creation of standardized acute dehiscence-type defect. (b) Implant insertion and exposed implant surface was treated with bone substitute grafting materials. (c) All defects were covered with Bio-Gide collagen membrane. (d) The flap was carefully closed by the mucoperiosteal flaps for submerged healing

After the 3-month healing period, the tooth extraction sites were reopened. Peeling off the mucoperiosteal flaps revealed the crest and buccal mandible bone. All drilling was done using copious amounts of sterile saline solution. After preparation of the implant cavity for implant insertion, a flat-head fissure bur with diameter 1.5 mm was used to create a standardized acute defects at the buccal bone of the implant cavity under constant irrigation with saline solution. The size of the standardized triangular-shaped defect is 6 mm apicocoronally, 12 mm mesiodistally and 2 mm buccolingually (see Fig. 1). Bone-level implants (diameter: 4.1 mm, length: 10 mm) were inserted into the defect sites according to the manufacturer instructions after saline solution rinse. The acute defects were repaired by the bone substitute following a randomized application sequence of TA, TB and BO bone-graft materials, while for the BC there was no bone-graft material repaired and the implant surface remained exposed. All defects were covered with Bio-Gide collagen membrane. The flap was carefully closed by the mucoperiosteal flaps for submerged healing. The surgical area was carefully sutured with absorbable suture line (Fig. 1). A soft diet was provided daily during the whole experiment period.

#### Sacrifice

The pigs were sacrificed at 3 and 6 months after implant insertion and ridge augmentation. Euthanasia was performed with an overdose of pentobarbital sodium. Subsequently, the implant and supporting tissues were harvested from the jaws and fixed in 10% buffered formalin for 4 weeks.

### Radiographic evaluation

Microcomputed tomography (micro-CT; Inveon MM; Siemens, Germany) was used to evaluate the volume of remaining materials after degradation and new bone formation. Images were acquired at an effective pixel size of 8.82 μm, voltage of 80 kV, current of 500 μA and exposure time of 1500 ms for each of 360 rotational steps. The volume of new bone formation and height of the bone augmentation sites were calculated using an Inveon Research Workplace (Siemens).

### Histological processing

The samples were series dehydrated in 50–99% alcohol solution. Finally, the dehydrated samples were embedded in autopolymerizing methyl methacrylate resin (Wako Pure Chemical Industries, Ltd., Japan). The resin-embedded specimens were oriented parallel to the longitudinal axis of the implant. One central section was cut in the buccolingual direction (300-μm thick) using a diamond saw (STX-202A; Shenyang Kejing Auto-instrument Co. Ltd., China), then adhered to the resin slides and ground to approximately 50-μm thickness. The ground slices were stained with methylene blue trihydrate and Van Gieson stain. The histological evaluation by a light microscope mainly focused on new bone formation, inflammatory reaction and especially, the degradation of materials and the response of surrounding tissues to the implant materials.

## Results and discussion

### Clinical examination

One mini pig was sacrificed due to excessive anesthesia during the implant operation. Although a soft diet was provided to all of the animals, rupture of the surgical wound was inevitable during the early healing phase. Exposure of the cover screw of the implant was observed in 20 of 80 implants; this occurred in all groups and with similar frequency. All other sites healed uneventfully. The movement and daily behavior of the tested animals were not affected. No animals died during the observation period.

### Micro-CT examination

#### Alveolar ridge height

**Figure 2:**
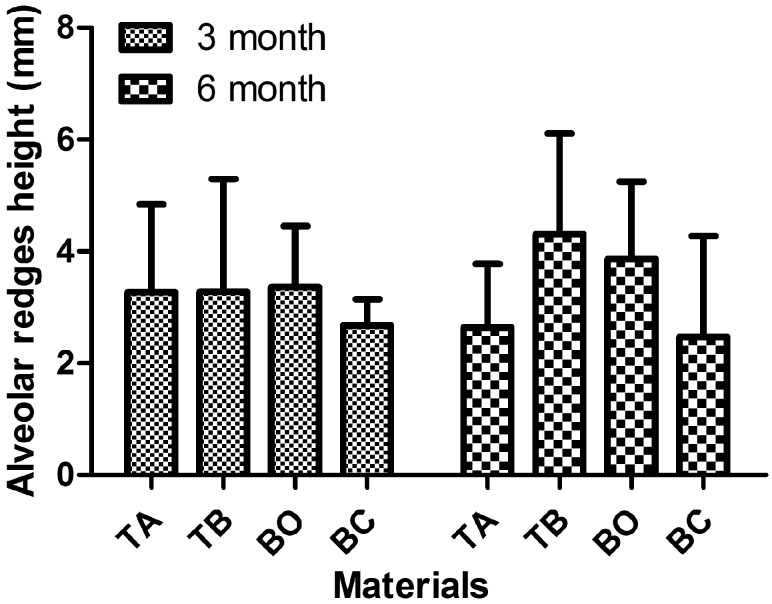
The alveolar ridge height of four tested groups after 3 and 6 months of healing. There was no significant difference among the test and control groups in either test period

Figure 2 shows the alveolar ridge height after 3 and 6 months of healing. This height is the vertical distance between the buccal crest at the middle of the implant and the bottom of the acute defect (6 mm from the implant platform). After 3 months of healing, the average alveolar ridge height was 3.27 ± 1.57 mm for group TA, 3.28 ± 2.02 mm for group TB and 3.37 ± 1.09 mm for group BO; group BC showed the lowest height of 2.68 ± 0.47 mm. After 6 months of healing, the average alveolar ridge height was 2.64 ± 1.13 mm for group TA, 4.31 ± 1.80 mm for group TB and 3.87 ± 1.38 mm for group BO; group BC again showed the lowest height of 2.48 ± 1.80 mm. There was no significant difference among the test and control groups in either test period. Although there was a slight decrease in vertical height for groups TA and BC from 3 to 6 months, and a slight increase for groups TB and BO, these differences were not statistically significant because of the large coefficient of variation.

Previous research reported that guided bone regeneration (GBR) can enable significant bone formation when used for treatment of dehiscence/fenestration defects around implants [15–17]. A recent systematic review showed that a satisfactory degree (ca. 80%) of defect filling of dehiscence defects around implants can be achieved using grafting materials regardless of their origin. Horizontal and vertical defects can be augmented predictably up to a width/height of ca. 3.7 mm using particulate grafting materials [18]. The bone augmentation height in the present study ranged from 2.68 to 3.47 mm, which corresponds to most clinical results [18].

A wide variety of grafting materials are available commercially, in various particle sizes and shapes. Clinical efficacy varies by defect geometry, augmentation technique and grafting materials. The results of the present study indicate similar clinical bone augmentation heights regardless of the origin or type of bone-grafting material. Many clinical studies have reported similar results. Troeltzsch *et al.* analyzed 7473 applications of graft materials in 6182 patients and confirmed that the reconstruction of peri-implant dehiscence defects was associated with the barrier membranes, rather than the graft materials, used [18]. Unlike clinical studies that investigated different defect types, GBR methods and barrier materials, the present study used standardized peri-implant bone defects that allowed direct comparison among the different graft-material groups [19]. The results of Buser *et al.* [21] and Jaffin *et al.* [22] showed that the placement of an implant can stimulated bone remodeling and bone maturation, which suggested that bone resorption may take place if the newly formed bone is not functionally loaded [20, 21]. In the present study, the newly formed bone remained stable during the 6-month observation period.

#### Bone volume density

**Figure 3:**
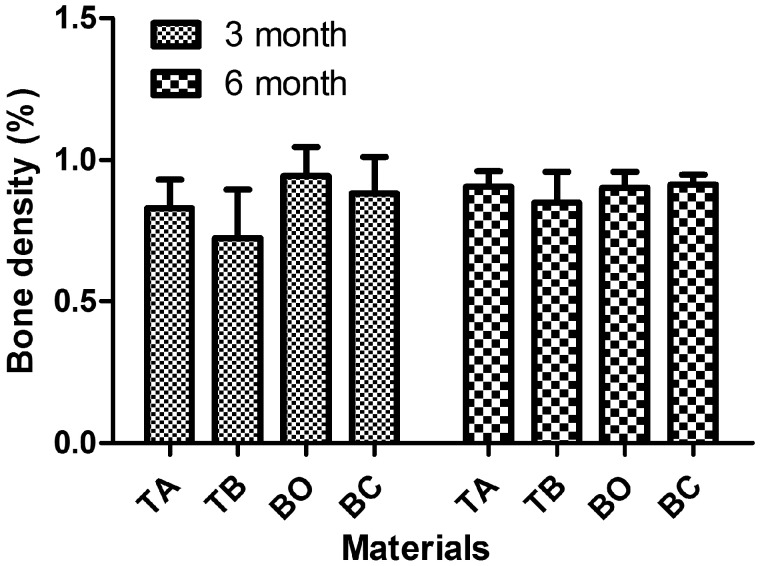
Bone volume density of four tested groups after 3 and 6 months of healing. The three types of materials used in the present study displayed similar bone volume density

The bone volume fraction is the ratio of the trabecular bone volume (BV) to the total volume (TV) of the area of interest (BV/TV). In addition to bone quantity, the success of an inserted implant strongly depends on the quality of the surrounding bone [22, 23]. Bone quality is associated with bone density and bone microarchitecture, which affect bone strength and fracture resistance [24]. There is a strong correlation between BV/TV and bone density, as assessed by micro-CT [25], and so the BV/TV ratio is a key parameter [26]. The four types of materials used in the present study displayed similar BV density (see Fig. 3). Although the TB material showed a slightly lower average value compared with the other three groups, there was no statistically significant difference.

#### Bone complexity

**Figure 4:**
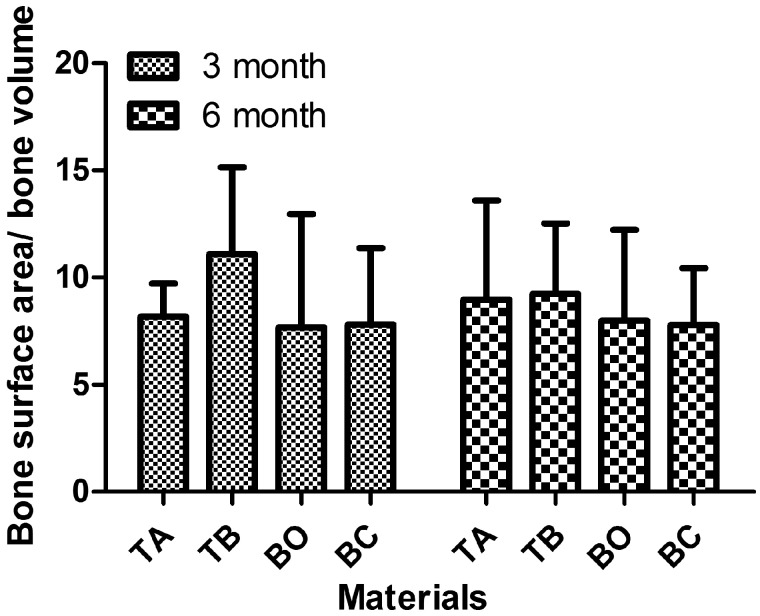
Bone complexity of four tested groups after 3 and 6 months of healing. The three types of materials used in the present study displayed similar bone complexity

Bone complexity is the ratio of the bone surface area (BS) to the total bone volume (BV) of the area of interest (BS/BV). It is another parameter for evaluating bone quality. The TB material had the highest average value of all of the groups, but there was no statistically significant difference among them (see Fig. 4).

### Histological evaluation

**Figure 5:**
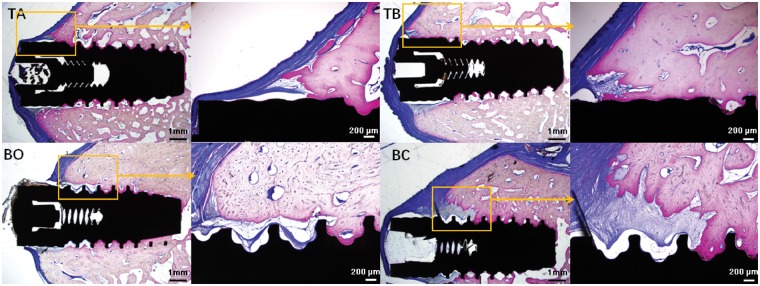
The histological results for the defect area after 6 months of healing. Similar histological results were observed for all of the groups in terms of bone regeneration and osseointegration. TA: the material was a mixture of HA and collagen. TB: the material is biomimetic mineralized collagen; BO : Bio-Oss bone-grafting material; BC: the blank control group contained only bone defects with a collagen membrane

Figure 5 shows the histological results for the defect area after 3 and 6 months of healing. All of the tested groups showed similar histological characteristics on the bone regeneration and osseointegration. The mature lamellar bone was direct contact with implant surface at the bone defect area and undefect area, irrespective of the graft material used. However, the bone-implant contact area is not consistent with the height of buccal bone. There is a wedge-shaped gap filled with soft tissue between the buccal alveolar ridge crest and implant for nearly all the tested groups, especially for the BC group. In the majority of cases, the graft particle can be integrated very well with newly formed bone; in BO group, some bone-grafting materials can be found surrounded by soft tissue in the buccal side of the defects. No remnants of the Bio-Gide collagen membrane were observed. However, for all the cases, the defect area was not completely repaired by new bone. Some threads can be exposed at the buccal coronal area of the implant in the BC group; this demonstrated that the model defect created in the present study was of critical size. The results of the present study resemble those of Zambon’s investigation, which used similar animal models [27]. Self-assembled type I bovine collagen and HA resemble natural MC. Preclinical trials showed that MC composed of biphasic calcium phosphate (HA/TCP) and collagen stimulated new bone formation during the reconstruction of deficient alveolar ridges in dogs [28, 29]. The limitation of present study is that there is large variation coefficient for the results due to the variation of experiment site, individual differences of animals and the limited sample size.

## Conclusions

A peri-implant bone defect model in mini pigs was developed to investigate the efficiency of self-assembled MC on peri-implant bone defect reconstruction in the dental clinic. The results show that self-assembled MC (Type B) stimulated new bone formation in the reconstruction of deficient alveolar ridges around a dental implant. Furthermore, it displayed excellent clinical operability compared with bone grafts without collagen. 

## Funding

This work was supported by Beijing Municipal Science & Technology Commission Projects (No. Z181100002018001).

*Conflict of interest statement*. None declared.

## References

[1] Buser D, Dula K, Hess D et al Localized ridge augmentation with autografts and barrier membranes. Periodontol 2000 1999;19:151–63. 10321222 10.1111/j.1600-0757.1999.tb00153.x

[2] Peñarrocha-Diago M, Gómez-Adrián MD, García-Mira B et al Bone grafting simultaneous to implant placement. Presentation of a case. Med Oral Patol Oral Cir Bucal 2005;10:444–7. 16264379

[3] Hoexter DL. Bone regeneration graft materials. J Oral Implantol 2002;28:290–4. 12498538 10.1563/1548-1336(2002)028<0290:BRGM>2.3.CO;2

[4] Sorní M, Guarinós J, García O et al Implant rehabilitation of the atrophic upper jaw: a review of the literature since 1999. Med Oral Patol Oral Cir Bucal 2005;10(Suppl. 1):E45–56. 15800467

[5] Esposito M, Grusovin MG, Coulthard P et al The efficacy of various bone augmentation procedures for dental implants: a Cochrane systematic review of randomized controlled clinical trials. Int J Oral Maxillofac Implants 2006;21:696–710. 17066630

[6] Bauer TW, Muschler GF. Bone graft materials. An overview of the basic science. Clin Orthop Relat Res 2000;371:10–27. 10693546

[7] Spin-Neto R, Stavropoulos A, de Freitas RM et al Immunological aspects of fresh-frozen allogeneic bone grafting for lateral ridge augmentation. Clin Oral Implants Res 2013;24:963–8. 22697457 10.1111/j.1600-0501.2012.02510.x

[8] Misch CM. Autogenous bone: is it still the gold standard? Implant Dent 2010;19:361. 20881805 10.1097/ID.0b013e3181f8115b

[9] Ruffieux K. A new syringe-delivered, moldable, alloplastic bone graft substitute. Compend Contin Educ Dent 2014;35:8–10. 25455149

[10] Sheikh Z, Hamdan N, Ikeda Y et al Natural graft tissues and synthetic biomaterials for periodontal and alveolar bone reconstructive applications: a review. Biomater Res 2017;21:9. 28593053 10.1186/s40824-017-0095-5 PMC5460509

[11] Shetty V, Han TJ. Alloplastic materials in reconstructive periodontal surgery. Dent Clin N Am 1991;35:521–30. 1879575

[12] Zhang W, Liao SS, Cui FZ. Hierarchical self-assembly of nano-fibrils in mineralized collagen. Chem Mater 2003;15:3221–6.

[13] Xu SJ, Qiu ZY, Wu JJ et al Osteogenic differentiation gene expression profiling of hMSCs on hydroxyapatite and mineralized collagen. Tissue Eng Part A 2016;22:170–81. 26529501 10.1089/ten.tea.2015.0237

[14] Liao SS, Cui FZ, Zhang W et al Hierarchically biomimetic bone scaffold materials: nano-HA/collagen/PLA composite. J Biomed Mater Res B Appl Biomater 2004;69:158–65. 15116405 10.1002/jbm.b.20035

[15] Retzepi M, Donos N. Guided bone regeneration: biological principle and therapeutic applications. Clin Oral Implants Res 2010;21:567–76. 20666785 10.1111/j.1600-0501.2010.01922.x

[16] Donos N, Mardas N, Chadha V. Clinical outcomes of implants following lateral bone augmentation: systematic assessment of available options (barrier membranes, bone grafts, split osteotomy). J Clin Periodontol 2008;35:173–202. 18724850 10.1111/j.1600-051X.2008.01269.x

[17] Chiapasco M, Zaniboni M. Clinical outcomes of GBR procedures to correct peri-implant dehiscences and fenestrations: a systematic review. Clin Oral Implants Res 2009;20:113–23. 19663958 10.1111/j.1600-0501.2009.01781.x

[18] Troeltzsch M, Troeltzsch M, Kauffmann P et al Clinical efficacy of grafting materials in alveolar ridge augmentation: a systematic review. J Craniomaxillofac Surg 2016;44:1618–29. 27622971 10.1016/j.jcms.2016.07.028

[19] Polimeni G, Koo KT, Qahash M et al Prognostic factors for alveolar regeneration: effect of a space-providing biomaterial on guided tissue regeneration. J Clin Periodontol 2004;31:725–9. 15312093 10.1111/j.1600-051X.2004.00542.x

[20] Donos N, Bosshardt D, Lang N et al Bone formation by enamel matrix proteins and xenografts: an experimental study in the rat ramus. Clin Oral Implants Res 2005;16:140–6. 15777322 10.1111/j.1600-0501.2004.01088.x

[21] Buser D, Ruskin J, Higginbottom F et al Osseointegration of titanium implants in bone regenerated in membrane-protected defects: a histologic study in the canine mandible. Int J Oral Maxillofac Implants 1995;10:666–81. 8530169

[22] Jaffin RA, Berman CL. The excessive loss of Branemark fixtures in type IV bone: a 5-year analysis. J Periodontol 1991;62:2–4. 2002427 10.1902/jop.1991.62.1.2

[23] Jemt T, Book K, Lindén B et al Failures and complications in 92 consecutively inserted overdentures supported by Brånemark implants in severely resorbed edentulous maxillae: a study from prosthetic treatment to first annual check-up. Int J Oral Maxillofac Implants 1992;7:162–7. 1398832

[24] Diederichs G, Link TM, Kentenich M et al Assessment of trabecular bone structure of the calcaneus using multi-detector CT: correlation with micro CT and biomechanical testing. Bone 2009;44:976–83. 19442610 10.1016/j.bone.2009.01.372

[25] Parsa A, Ibrahim N, Hassan B et al Bone quality evaluation at dental implant site using multislice CT, micro-CT, and cone beam CT. Clin Oral Implants Res 2015;26:e1–7. 10.1111/clr.12315 24325572

[26] Parfitt AM, Drezner MK, Glorieux FH et al Bone histomorphometry: standardization of nomenclature, symbols, and units. Report of the ASBMR Histomorphometry Nomenclature Committee. J Bone Miner Res 2009;2:595–610. 10.1002/jbmr.5650020617 3455637

[27] Zambon R, Mardas N, Horvath A et al The effect of loading in regenerated bone in dehiscence defects following a combined approach of bone grafting and GBR. Clin Oral Implants Res 2012;23:591–601. 22092957 10.1111/j.1600-0501.2011.02279.x

[28] Pagni G, Pellegrini G, Giannobile WV et al Postextraction alveolar ridge preservation: biological basis and treatments. Int J Dent 2012;2012:1. 10.1155/2012/151030 PMC3378971 22737169

[29] Wang RE, Lang NP. Ridge preservation after tooth extraction. Clin Oral Implant Res 2012;23:147–56. 10.1111/j.1600-0501.2012.02560.x 23062139

